# Invasive Liver Abscess Syndrome in the Immunocompromised: A Case Report

**DOI:** 10.1002/ccr3.70108

**Published:** 2025-01-10

**Authors:** Amey Joshi, Eki Wari, Harith Ghnaima, Georgette Nader, Surya Chennupati, Nagham Jafar

**Affiliations:** ^1^ Department of Internal Medicine Sparrow Hospital—Michigan State University East Lansing Michigan USA; ^2^ Sparrow Hospital—Michigan State University East Lansing Michigan USA

**Keywords:** Hypervirulent *klebsiella pneumoniae*, invasive liver abscess syndrome, splenectomy, uncontrolled diabetes mellitus

## Abstract

Invasive liver abscess syndrome due to hypervirulent *Klebsiella pneumoniae* poses significant mortality risk, particularly in immunocompromised patients. Early recognition in non‐endemic regions is crucial for prompt antibiotic therapy and source control, highlighting the need for increased suspicion and aggressive management of this rare disease to improve patient outcomes.

## 1 Introduction

Invasive liver abscess syndrome (ILAS) caused by *Klebsiella pneumoniae* ( *K. pneumoniae* ) is a rare clinical condition characterized by bacteremia and metastatic infections with disseminated abscesses [1]. Extrahepatic metastatic infections may result in severe complications and represent a poor prognosis if not appropriately treated [2]. The invasive nature of the disease is due to certain subtypes of *K. pneumoniae* with virulence characteristics such as hypermucoviscosity phenotypes [1].

ILAS is prevalent mostly in Asian and African countries, but only a handful of cases have been reported in Western countries [3]. In immunocompetent individuals, these infections usually respond to an extended course of antibiotics, and mortality rates range from 4% to 11% [2]. However, in the context of immunocompromised individuals, the mortality rates can be significantly higher [4]. Herein, we present a case of ILAS caused likely by the hypervirulent mucus phenotype of *K. pneumoniae* in the background of multiple risk factors, including a history of splenectomy and newly diagnosed uncontrolled type 2 diabetes mellitus.

## 2 Case History

A 34‐year‐old male with a history of immigration from the Republic of Congo presented with complaints of abdominal pain and shortness of breath for 2 days. He had a past medical history of significant recurrent alcoholic pancreatitis leading to splenic vein thrombosis and splenectomy. He was noted to be febrile (102.2 F), tachycardic, tachypneic, and hypoxic, requiring 2 L of oxygen supplementation.

## 3 Methods

**FIGURE 1:**
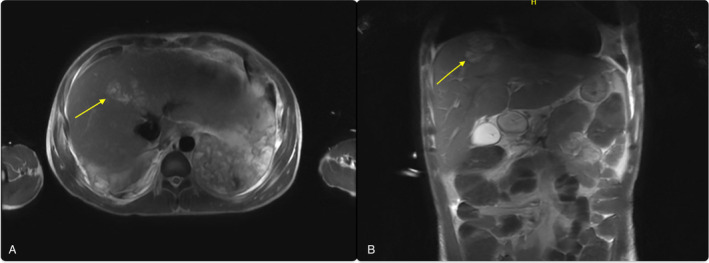
Magnetic resonance imaging (A) Coronal view (B) Sagittal view, showing liver abscess.

**FIGURE 2:**
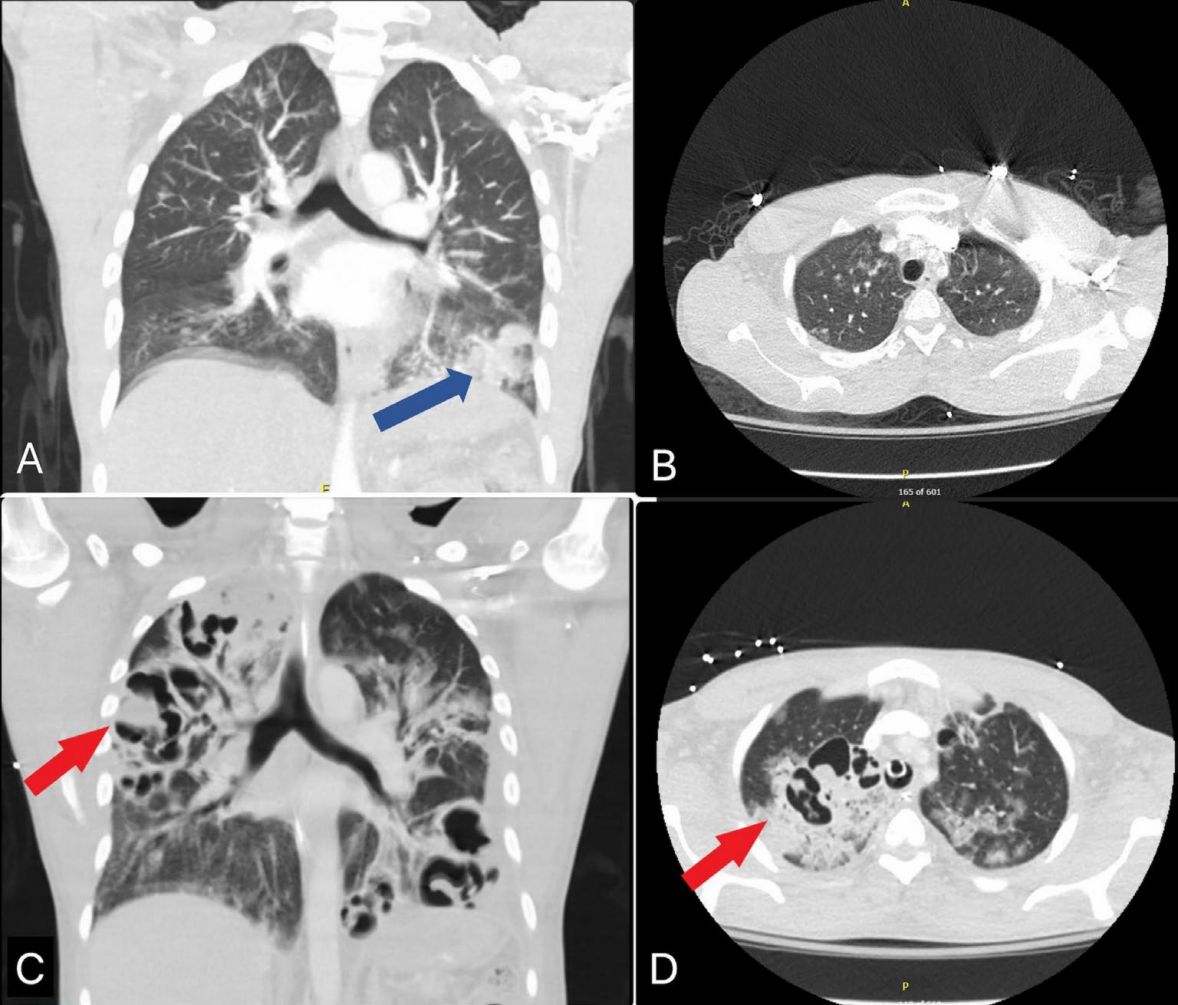
CT imaging of the chest (A) Sagittal view, and (B) Coronal view showing disease process at presentation with large left lower lobe consolidation (Blue arrow); (C) Sagittal view, and (D) Coronal view showing (red arrows) areas of cavitations and multifocal pneumonia.

His laboratory investigations were notable for high anion gap metabolic acidosis, elevated blood sugars, glycosylated hemoglobin of 19%, and ketonuria with positive beta‐hydroxybutyrate. His initial CT imaging revealed bilateral lower lobe consolidations and also an abscess in the liver (Figures 1 and 2). He was started on an insulin infusion and fluid resuscitated for diabetic ketoacidosis and anti‐bacterial coverage with Piperacillin‐Tazobactam for the management of pneumonia and liver abscess. The patient tested negative for HIV, hepatitis B, and C, TB by quantiferon analysis, *Neisseria gonorrhoeae* , MRSA, COVID‐19, and EBV.

Due to worsening hypoxia and increased work of breathing, the patient was intubated 2 days into his admission. Bronchoscopy with serial washings was done, and bronchoalveolar cultures were positive for *K. pneumoniae* growth. These were also negative for acid‐fast bacilli and fungi. Ultrasound‐guided drainage of the liver abscess was done, and the drain was left in situ. The liver‐drained abscess culture was also positive for *K. pneumoniae* . The patient continued to be febrile with worsening of leukocytosis with a predominant left shift (18,000/mm^3^; normal: 4500‐11,000/mm^3^). Due to his worsening clinical condition, antibiotics were adjusted to meropenem and linezolid with antifungal coverage with micafungin. Repeat CT imaging revealed multiple lung cavitations and abscesses (Figure 2). The patient's oxygen requirements continued to increase on mechanical ventilation. Due to the multiplicity and extensiveness of the lung abscesses, surgical options were very limited and deferred. Due to increasing suspicion of hypervirulent *K. pneumoniae* with high mucus phenotype, antibiotics were further escalated to include tobramycin and rifampin.

Despite multiple bronchial washings, drainage of liver abscess, and 27 days of antibiotic therapy, the patient's condition continued to decline. Eventually, he was noted to be in distributive shock secondary to severe sepsis, requiring three pressor support with phenylephrine, norepinephrine, and vasopressin. His course was further complicated by renal failure, and continuous renal replacement therapy was attempted. The patient demised in spite of maximum support and medical therapy.

## 4 Conclusion

This case highlights the extremely high mortality in ILAS with multiple risk factors. Despite aggressive attempts at source control and antibiotic regimens, due to the significantly immunocompromised state and extensiveness of the disease, our patient deteriorated rapidly, leading to mortality. Timely diagnosis and a low threshold of suspicion for these rare subtypes/phenotypes of *K. pneumoniae* , especially in non‐endemic regions like the United States, is crucial to initiate prompt treatment and source control.

## 5 Discussion

This case details ILAS in a 34‐year‐old man with substantial medical comorbidities, including newly diagnosed uncontrolled diabetes (hemoglobin A1c > 19%) and a remote history of splenectomy. Due to the extensive damage to the liver by the invasive bacterial species, he also developed hypoproteinemia and hypoalbuminemia during the hospital course, worsening his immunological status. These put him in an immunocompromised state, worsening the trajectory of his illness, and leading to mortality despite early identification of the offending organism and aggressive management with multiple antibiotics regimens. Furthermore, this case presents a unique challenge regarding source control due to rapidly progressing disseminated abscesses involving the liver and multiple segments of both lungs within a very short period. The lesions' severity and anatomical distribution made abscess drainage a challenging and non‐applicable option.

Most community‐acquired *K. pneumoniae* infections cause pneumonia and urinary tract infections; however, a rare and more distinct invasive syndrome has been associated with this infection in the last two decades [5, 6]. ILAS, defined by the development of one or more abscesses inside the liver, was found to be caused by a hypervirulent strain of *K. pneumonia* that had serious and often life‐threatening complications. These infections have more commonly been reported in the Southeast Asian subcontinent and their incidence over the last two decades has only been increasing. In a retrospective study conducted on patients in Taipei with liver abscesses, 78% of the abscesses were caused by *K. pneumoniae* [6].

Hypervirulent strains often result in more extensive infections, resulting in pyogenic liver abscesses, necrotizing fasciitis, meningitis, myositis, and endophthalmitis [7]. Serotypes K1 and K2 are most often associated with hypervirulent strain. Unlike classical *K. pneumoniae* , which are nosocomial in nature, hypervirulent strains are most commonly community‐acquired [8]. These are usually more invasive and have a tendency to become disseminated. The bacterium's capacity to proliferate and elude the host immune system is greatly enhanced by the acquisition of large plasmids that enhance capsule formation and siderophore encoding, which are genetic elements that are partially responsible for this hypervirulence [7].

Notably, compared to those with liver abscesses caused by other organisms, individuals with liver abscesses from *K. pneumoniae* had a higher prevalence of diabetes mellitus (66% vs. 19%) [6]. Regardless of the microbiological source, diabetic patients, such as in the present case, had longer fever durations and required prolonged hospital stays [6]. This is likely because hyperglycemia can increase the risk of infection in patients with diabetes through several different processes, including hyperglycemia‐induced delayed chemotactic response, phagocytosis, and neutrophil activity [9].

Due to the infections' rapid progression and significant resistance profile, the diagnostic strategy for these infections is still complicated and requires timely microbial culture, sensitivity testing, and molecular approaches to detect particular resistance genes and virulence components [10]. Patients often endorse nonspecific symptoms of fever, chills, and abdominal pain [11]. Other potential findings include elevated transaminases and CRP of greater than 20 [2]. Patients with diabetes mellitus who are noted to have *K. pneumoniae* bacteremia, meningitis, or endophthalmitis should be evaluated for a potential liver abscess [1]. Abdominal CT is the preferred diagnostic study compared to ultrasound [12]. Abscesses secondary to *K. pneumoniae* aggregate in a single lobe and are solid in characteristic and multilocular [12].

Antimicrobial treatment is usually tailored according to the sensitivity pattern of the bacteria. A third‐generation cephalosporin is often used; however, ampicillin‐sulbactam, aztreonam, or quinolone can be considered. Carbapenems can be used with strains that produce extended‐spectrum beta‐lactamases (ESBL) [1]. Rifampin has also been shown to have anti‐mucoviscous activity against hvKp, and some cases have documented clinical improvement in ILAS with a combination of meropenem and rifampin [13]. Some case reports have used either a combination or sequential use of the above antibiotics with varying success rates [13, 14]. For diabetic patients, strict glycemic control is also an important part of management. Percutaneous drainage can be done in the setting of limited abscesses to improve clinical response [15]. The *K. pneumoniae* isolated in this patient was resistant only to ampicillin; thus, multiple antibiotic regimens were attempted, including Piperacillin‐Tazobactam, meropenem, rifampin, and tobramycin.

It is important to recognize the pattern of dealing with liver abscess and pneumonia at time of diagnosis. Promoting the physician to suspect highly resistant strains that likely require an early and aggressive antibiotic regimen and possibly surgical drainage of the abscess to achieve better source control as this might change the outcome.

## Author Contributions

**Amey Joshi:** conceptualization, data curation, formal analysis, investigation, methodology, supervision, visualization, writing – original draft, writing – review and editing. **Eki Wari:** conceptualization, investigation, project administration, validation, visualization, writing – original draft, writing – review and editing. **Harith Ghnaima:** investigation, project administration, supervision, visualization, writing – original draft, writing – review and editing. **Georgette Nader:** investigation, methodology, project administration, supervision, validation, writing – original draft, writing – review and editing. **Surya Chennupati:** conceptualization, data curation, supervision, visualization, writing – original draft, writing – review and editing. **Nagham Jafar:** conceptualization, formal analysis, investigation, methodology, project administration, resources, software, supervision, writing – review and editing.

## Consent

Written informed consent was obtained from the patient's family to publish this report as the patient was critically ill and unfortunately demised. This case report has been completely anonymized.

## Conflicts of Interest

The authors declare no conflicts of interest.

## Data Availability

The data supporting the findings of the present study are available from corresponding author upon request.
